# Gender, Parenthood and Wage Differences: The Importance of Time-Consuming Job Characteristics

**DOI:** 10.1007/s11205-016-1271-z

**Published:** 2016-02-15

**Authors:** Charlotta Magnusson, Magnus Nermo

**Affiliations:** 1 Swedish Institute for Social Research (SOFI), Stockholm University, 106 91 Stockholm, Sweden; 2 Department of Sociology, Stockholm University, 106 91 Stockholm, Sweden

**Keywords:** Gender inequality, Wages, Occupational prestige, Fatherhood premium, Job characteristics

## Abstract

Using data from the Swedish Level of Living Survey (2000, 2010), we investigate how the gender wage gap varies with occupational prestige and family status and also examine the extent to which this gap is explained by time-consuming working conditions. In addition, we investigate whether there is an association between parenthood, job characteristics and wage (as differentiated by gender). The analyses indicate that there are gender differences regarding prestige-based pay-offs among parents that are partly explained by fathers’ greater access to employment characterized by time-consuming conditions. Separate analyses for men and women demonstrate the presence of a marriage wage premium for both genders, although only men have a parenthood wage premium. This fatherhood premium is however only present in high-prestigious occupations. Compared with childless men, fathers are also more advantaged in terms of access to jobs with time-consuming working conditions, but the wage gap between fathers and childless men is not explained by differences in access to such working conditions.

## Introduction

The growing number of women in both higher education and high-skilled occupations in recent decades signifies important steps toward more gender equality. However, this trend has not erased the persistent gender wage gap in Sweden and elsewhere (e.g., Boye et al. 2014; Blau and Kahn 2007; OECD 2012). Today, the unadjusted gender wage gap in Sweden is around 15 % (Boye et al. 2014). It is also clear that the gender wage gap in Sweden is more salient (1) among the highly educated (Evertsson et al. 2009), (2) in the upper end of the wage distribution (Albrecht et al. 2003; Bihagen et al. 2014a), (3) in high-prestige occupations (Magnusson 2009). One factor that is frequently cited as important to understanding the persistent gender wage gap involves gender differences in family responsibilities (e.g., Boye et al. 2014; Williams 2010). Thus, it is argued that the skewed division of unpaid labor by gender in families is both indirectly and directly related to the gender wage gap. Consistent with the foregoing, previous research generally indicates that men’s careers and wages are positively related to having a family, whereas the same seems to hinder women’s careers (Budig and England 2001; Correll et al. 2007; Hinze 2000). Even if recent research suggest that marriage is positively related to both men’s and women’s wages (e.g., Budig and England 2001; Killewald and Gough 2013; Petersen et al. 2014), the marriage premium is larger for men than for women (e.g., Petersen et al. 2014). There is also evidence suggesting a negative relationship between wages and motherhood (Budig and England 2001; Budig and Hodges 2010; Gough and Noonan 2013). However, these studies—and the data they employ—are mainly American, whereas Nordic studies based on Finnish (Gash 2009) and Norwegian data (Petersen et al. 2014) do not find a similar negative relationship between motherhood and wages. For men most studies, both American and European ones, show that fatherhood is positively associated with wages (Boschini et al. 2011; Glauber 2008; Killewald 2013 1; Petersen et al. 2014, Polachek 2004; Smith Koslowski 2011).

Even if these findings are somewhat contradictory, it seems clear that the association between family status and wage is different for women than it is for men. However, although we know that having a family affects men’s and women’s labor market outcomes differently, we know relatively little about whether and how the magnitude of these effects differs with occupational prestige level. Thus, we lack knowledge about whether the role of family for the gender wage gap varies by occupational prestige. We also lack knowledge about the within-gender association among parenthood, job characteristics and wage, and/or whether this association varies with occupational prestige.

One potential explanation for the wider gender gap among workers in high-prestigious occupations may be that these occupations are more likely to include high demands on “loyalty” to the organization and constant availability (e.g., Williams 2000; Acker 1990) e.g., working non-agreement overtime, taking part in organizational arrangements outside regular hours and travelling on business. Such demands are often difficult to combine with an actual, or by the employer assumed, responsibility for the family (Magnusson 2010; Blair-Loy 2003; Williams 2000: 2010; Goldin 2014). According to the gender skewed division of family duties it is reasonable to assume that among those workers with family obligations, it is more difficult for women than men to have wage promoting job characteristics in high-prestigious occupations (Magnusson 2010; Blair-Loy 2003). In other words, it is reasonable to assume that women’s greater family responsibilities have a stronger negative effect on wages and career opportunities in high-prestigious positions than in low-prestigious occupations. Thus, to obtain the best possible wage growth in high-prestige occupations, the employee must be constantly available to the employer to work extra hours and to take part in activities outside normal working hours (e.g., Blair-Loy 2003). This assumption about what is here defined as time-consuming job characteristics is supported by a previous Swedish study indicating that gender wage differences among parents in high-prestige occupations is largely attributed to gender differences in access to employment characterized by time-consuming job conditions, such as over-time work, business travel and unpaid overtime (Magnusson 2010). However, to our knowledge, no previous study has examined the importance of time-consuming and wage promoting job characteristics to understanding both the gender wage gap at different levels of occupational prestige and the within-gender association between parenthood and wages, that is, whether differences in time-consuming work could account for potential wage premium or penalties for parenthood within-gender.

Here we pool data from the two latest waves of the Swedish Level of Living Survey (LNU 2000 and 2010) that include rich information that is rarely found in large surveys on both working conditions and family status. Using these data, we first make a significant contribution to the literature by analyzing (a) the association between time-consuming job characteristics and the gender wage gap at different levels of occupational prestige separately for (1) single persons, (2) couples with children, and (3) couples without children; we also contribute to the literature by analyzing (b) whether there is a within-gender association between parenthood, time-consuming job characteristics and wages, and if so, (c) whether this premium (or penalty) varies with occupational prestige.

By studying the within-gender association between wages and job characteristics, we are able to investigate whether the distribution of these job characteristics differs between mothers and non-mothers and between fathers and non-fathers. For instance, it is possible that married or cohabiting fathers have greater access to these career- and wage-promoting job characteristics, which in turn partly explain the marriage and fatherhood wage premiums found for men in previous research.

This paper is organized as follows. First, we briefly describe gender differences in the Swedish labor market and discuss relevant theories and the previous research on the association between the gender wage gap and family responsibilities. The empirical segment of the paper includes analyses of the association between time-consuming job characteristics and the gender wage gap at different levels of occupational prestige. These analyses are performed separately for the three family statuses discussed above and also separately for men and women. The paper ends with a summary and discussion of the reported findings.

## Background

### The Swedish Case

For decades, Sweden has been one of the most gender equal countries in the world, and Sweden’s social policy has for long been characterized by a so-called dual earner model in which both parents are expected to work; moreover, Swedish family policies facilitate this model by offering publicly subsidized child care and paid parental leave, among other incentives (Korpi 2000). This model has been extremely successful in terms of facilitating women’s labor force participation; since the 1980s, roughly half of the Swedish labor force has consisted of women. Thus, the female employment rate is high—approximately 77 % of all women between the ages of 20 and 65 were gainfully employed in 2013. Moreover, during recent decades, women’s labor market activity has increased in highly skilled occupations, in particular, and women have higher levels of education than men, on average (Statistics Sweden 2007/2008). Nonetheless, as discussed above, differences remain between men and women in the Swedish labor market and with respect to unpaid labor. Women continue to perform the majority of unpaid family work and are more likely than men to work part-time. Thus, many Swedish women reduce their work time significantly when they become mothers, whereas men’s paid work time is almost unaffected by fatherhood (Boye and Evertsson 2014). Similarly, among parents with young children, the time that women spend performing unpaid housework is twice as high as that of men (Boye 2014). Mothers also use approximately 75 % of all parental leave days and approximately 65 % of all care days for sick children (Duvander and Viklund 2014).

Given this skewed gender division of family responsibilities and despite subsidized child care and other gender egalitarian social policies, women might find it more difficult than men to combine having a family and holding a well-paid position. In particularly in highly skilled occupations typically characterized by time-consuming working conditions, such as constant availability, frequent overtime and business travel. It is also worth noting that gender-egalitarian family policies have been criticized for contributing to a larger under-representation of women in supervisory positions and for increasing the gender wage gap, especially in highly skilled occupations (e.g., Mandel and Semyonov 2005). Subsidizing child care, for example, is a service that makes it possible for both parents to be active in the labour market and to combine work and family life, but the child care facilities are, as Petersen et al. (2014) assert, open relatively short hours, typically from 6.30 a.m. to 5.30 p.m. (or shorter). This is an important service for parental employment but does not facilitate careers for parents in high-paying professional jobs (Petersen et al. 2014). Negative effects of parental leave and taking care of sick children might of course vary between positions and occupations. Thus, child care policies facilitates parents (or in general women’s) possibilities to be gainfully employed, but do not in practise make it easier for women to hold positions characterized by time-consuming work.

The Swedish family policies may also affect men’s career opportunities. Even if the use of parental leave and the division of unpaid work still is uneven, many Swedish fathers take parental leave—especially highly educated fathers (Social Insurance Report 2011). Thus, on average Swedish fathers differ in this respect compared to fathers in, e.g., the US and most European countries. The association between fatherhood and wages may accordingly also be different in Sweden than in other countries (e.g., Killewald 2013; Prince Cooke 2014). However, as mention above, previous research implies that there is a positive relationship between fatherhood and wages also in Sweden (cf. Bihagen et al. 2014b). The association between fatherhood and career outcomes among Swedish men is yet not fully disentangled, especially not to what extent it varies according to occupational prestige. The present paper is thereby an attempt to increase our knowledge regarding this issue.

As mentioned previously the relationship between wages and motherhood in Sweden and other Nordic countries is not clear-cut. While studies, based mainly on American data, report a negative relationship between wages and motherhood (e.g., Gough and Noonan 2013) studies using data from Finland and Norway (Gash 2009; Petersen et al. 2014) report no or only a small motherhood wage penalty. Previous research indicates a large gender wage gap in Sweden but since there is not clear empirical evidence of a motherhood wage penalty the disadvantages in wages seems to affect all women and not especially mothers. Instead recent Swedish studies indicate a wage premium for parents, although this premium is significantly higher for men than for women (e.g., Bihagen et al. 2014b; Boschini et al. 2011).

To sum up, the somewhat divergent associations between family status and wages together with high female labour supply and large gender wage gap makes Sweden an interesting country to study the association between wages and job characteristics for both men and women.

### The Relationship Between Gender, Family Status, High-Skilled Occupations and Wage

As indicated above, gender differences in family and household responsibilities are frequently posited to explain gender inequality in the labor market. In line with human capital theory, the explanation is that the association between women’s family status and wage is explained by gender differences in individual productivity stemming from the gendered division of paid and unpaid work (Becker 1967, 1991; Glauber 2008). In addition, some scholars argue that reduced human capital due to absence from the labor market may be even more salient for highly educated women (e.g., Anderson et al. 2002), which is supported by studies indicating that the wage penalty for motherhood rises as the level of education increases (Waldfogel 1997). However, other American studies indicate the opposite, i.e., that the motherhood wage penalty is larger for women earning lower wages than for women earning higher wages (Budig and Hodges 2010). This finding is further supported by a Swedish study indicating that the association between income at age 45 and the number of children is negative for women without a university degree, while it is positive for university-educated mothers born in the 1950s and 1960s (Boschini et al. 2011). Corresponding findings for men indicate a clearly positive association between fatherhood and income for university-educated (Boschini et al. 2011) and college-educated men in professional occupations (Hodges and Budig 2010) and among high-earning men (Prince Cooke 2014). Similarly, a Swedish study shows that fathers are more likely than childless men to hold a top wage position in the labor market (Bihagen et al. 2014b). These prior empirical findings indicate a premium for fatherhood which tends to be greater in high-prestigious occupations while the results for motherhood are inconclusive and points both on premiums and on penalties.

By contrast, there are also demand-side explanations that might be important to understanding the gender wage gap in high-skilled occupations, in particular. According to gendered organization theory, the cultural assumptions of men and women influence and arrange organizational rewards and determine the positions men and women are allocated in the organizational structure (Acker 1990; Glauber 2008). Some scholars, such as Glass (1999), argue that employers construct profiles of promising and competent employees and that the ideal employee profile is married but without primary responsibility for the unpaid work in the family because such an employee is more likely to commit to time-consuming working conditions. In general, this profile means that there is an imminent risk that women and men are valued and treated differently by employers as long as women are—or are assumed to be—primarily responsible for the family. Of course, cultural assumptions (such as the above) may also affect within-gender differences, such as between single childless men and married fathers. Thus, one possible explanation for male marriage and parenthood wage premiums is that employers perceive fathers as more stable, responsible and committed than childless men (cf. Correll et al. 2007). The importance of responsibility and stability could also vary according to prestige level where such characteristics may be more important in high-prestigious occupations. In the classical work of Kanter from (1977) she describes a homo social process in an organization where the “managerial group” is a closed circle of selected men. Kanter asserts that men in higher positions, to a large extent, tend to reproduce themselves. Homo social processes are principally strategies to deal with organizational uncertainty. Thus, to avoid uncertainty managers largely select subordinates that act like themselves and whom they therefore can trust. Such system does not only shut women out, it could also normalize a certain kind of masculinity in organizations (Collinson and Hearn 2005). Married fathers in high-prestigious occupations could, according to this reasoning, be more advantaged than both women and childless men.^2^

To sum up, parenthood seems to have different effects on women’s and men’s wages, and women seem to be particularly disadvantaged in high-skilled occupations. This impact in turn might be related to the fact that wages in these occupations are determined to a large extent by time-consuming job characteristics that are difficult to combine with family responsibilities, (cf. Blair-Loy 2003; Nsiah et al. 2013; Williams 2000). Thus, given education and skills, these job characteristics are therefore likely to restrict women more than men from holding highly paid positions in the labor market (Rutherford 2001; Blair-Loy 2003). For instance, Magnusson (2010) report that gender differences with respect to time-consuming job characteristics account for a substantial share of the gender gap in returns from occupational prestige among cohabiting fathers and mothers. Likewise, Goldin (2014) asserts that a substantial part of the remaining gender wage gap might be ascribed to the fact that employers tend to reward individuals who work long hours and who are available most of the time. Given that mothers are seen as having the main responsibility for family-life they may be offered (or avoiding) time-consuming work to a lesser (larger) extent than fathers which in the end result in lower wages. However, as the amount of time-consuming work in the occupation vary according to occupational prestige the importance of time-consuming work for the gender wage gap are supposed to be greater in high-prestige occupations. Furthermore, how time-consuming work characteristics are distributed within-gender is, to our knowledge, unknown. Thus, we know very little of whether or not it is within-gender differences in time-consuming work and if so, whether or not this could account for potential wage premium or penalties for parenthood within-gender.

Another important factor for determining wages is whether the employee holds a supervisory or managerial position. Many prior studies have shown a gender difference in supervisory positions to women’s disadvantages (e.g., Bihagen et al. 2014a; Smith 2002). Even given the same level of occupational prestige there may thus be gender differences in access to managerial positions. Previous research indicates that the gender difference in being promoted to managerial positions is partly related to family status (see e.g., Bygren and Gähler 2012; Granqvist and Persson 2005). For instance, Bygren and Gähler (2012) report a strong association between family status and the likelihood that a woman will hold a managerial position. These authors find no gender difference in this respect between childless women and men. Instead, the gender difference was explained by the fact that men’s chances for promotion increased when they became fathers, whereas the same chances for women were unaffected by motherhood.

Taken together previous research indicates that parenthood has a different impact on women’s and men’s wages and that the importance of family role for the gender wage gap varies by occupational prestige. The present study contributes to the literature by studying the extent to which the substantial gender wage gap found in high-prestige occupations is explained by gender differences in wage-promoting but time-consuming employment conditions. Furthermore, we also analyze the within-gender association between parenthood, time-consuming characteristics and wage and whether this association varies with occupational prestige. Thus, by studying women and men separately we are able to investigate whether mothers are more disadvantaged than childless women in terms of access to jobs with these characteristics and whether the distribution of access to jobs with these characteristics differs among fathers and childless men.

## Methods

### Data

The data are from the Swedish Level-of-Living Survey (LNU), which is a national representative survey of 0.1 % of the Swedish population between the ages of 18 and 75. The LNU has been conducted six times since 1968. In the present study, we use data from the years 2000 and 2010, the two latest waves of the LNU. In LNU 2000, 5142 respondents participated, with a response rate of 76.6 %. The corresponding response rate in 2010 was 61.7 %. Specifying models separately for singles, couples without children and couples with children, and estimating separate analyses for mothers/fathers and non-fathers/non-mothers requires a large sample. Therefore, to increase the number of respondents, we pool the two latest waves of the LNU, which yields a total sample of 4274 employees aged 20–65 working at least 10 h per week.^3^

### Dependent Variables

The dependent variable in the analyses is the logarithm of *hourly wage*. Using a logarithmic dependent variable in an OLS regression, a change by one unit in the independent variable produces a certain percentage change in the dependent variable (Allison 1999). The following estimation is used to calculate the percentage change: 100(exp(b) − 1). When information on hourly wages is missing, other types of pay (such as daily, weekly or monthly pay) have been recalculated into hourly wages. The wage variable includes bonuses, piece-rate, other earnings benefits, and compensation for overtime (see le Grand 1991 for a further description). Because we pool two waves of the LNU, the hourly wage for respondents in 2000 was standardized as 2010 prices using the consumer price index.

### Explanatory Variables

The skill level of occupations is defined here by their *Occupational prestige,* which is based on Treiman’s Standard International Occupational Prestige Scale (SIOPS). The SIOPS is based on national populations’ subjective valuations of occupations from 60 countries, integrated into an international scale (Treiman 1977). Prestige, which is based on evaluations, captures a structural order of occupations by their general desirability (Goldthorpe and Hope 1974). Studies of prestige have shown that these ratings are stable over time and place, and that occupational prestige is highly correlated with both education and earnings (Wegener 1992) and that prestige could also be understood as a measure of occupational skill level. The prestige variable used here is continuous and ranges from 13 to 78. Examples of high-prestige occupations include physicians, university professors, lawyers and engineers, and examples of low-prestige occupations include cleaners, construction laborers and garbage collectors. Occupations are classified according to the International Standard Classification of Occupations (ISCO-88).

The variable *Woman* is coded one if the respondent is a woman and zero if a man. The variable *women* *×* *prestige* is an interaction term between women and prestige and measures whether there is any gender difference in wage return for attained occupational prestige. The variable *Parent* indicates whether the respondent is a parent and includes all respondents with children in the household and all respondents who have biological or adopted children who do not live in the respondent’s household at the time of the interview (e.g., adult children). Adult children are also included because it is plausible that parenthood involves a long-term effect on wage growth, particularly on women’s wages because of longer labor market interruptions and periods of part-time work (cf. Budig and England 2001). *Number of children* indicates the number of children living in the respondent’s household at the time of the interview. It is also plausible that gender differences in work characteristics that are difficult to reconcile with family responsibilities are greater when children are young and the need for care is more intense. We therefore also control for whether at least one of these children is under 12 years of age, *Young children*. To capture family responsibilities we also include a control for *Housework* which measures the number of hours during a normal week the respondents spends on buying groceries, cooking, washing dished, cleaning and laundering.

The variables *Business travel, Working overtime, Unpaid overtime work,* and *Number of subordinates* are included as measures of time-consuming job. These characteristics are all not only difficult to combine with having the main responsibility for family life but also positively related to wages (Magnusson 2010). *Business travel* is included as a continuous variable measuring the number of overnight accommodations yearly that are related to work. *Working overtime* is a dummy variable and indicates how often the employee works overtime (0 = never/very seldom and 1 = often/several days a week). *Unpaid overtime work* indicates whether the employee is expected to work overtime without extra compensation. If the employee is not compensated for overtime, hourly wages are normally higher because the compensation is already included in the normal salary. In other words, unpaid overtime is normally positively associated with wages. *Number of subordinates* is a categorical variable that separates having 0, 1–5, 6–10, and 11 or more subordinates (cf. Bygren and Gähler 2012).

*Education* (years) and *work experience* (years) and *work experience squared* are used to measure respondents’ human capital. Controlling for work experience is important because employees’ experience is associated with career development. *Age*, *sector* and *proportion of women in the occupation* are included as controls in some of the analyses. The age of employees influences the likelihood of being married/cohabiting and the likelihood of being a parent and is therefore important to take into account. *Sector* is a dummy in which public sector is coded 1. *Proportion of women in the occupation* is measured as the percentage of females in each occupation and based on data from the 2010 employment register (Statistics Sweden). By taking sector and occupational gender composition into account, we ensure that the gender wage gap by occupational prestige is not based on an effect of the skewed distribution of men and women across both sectors and occupations.

## Results

The descriptive statistics presented in Table 1 indicate that the majority of the respondents are cohabiting/married and have children. It is also clear that more women than men in the sample are parents. Women tend to perform more housework per week than men. There are only marginal gender differences with respect to years of education, but men have slightly more work experience, on average. A majority of women are employed in the public sector. The corresponding figure for men is approximately 30 %. The statistics in Table 1 also indicate that there are significant gender differences in the distribution of time-consuming work to women’s disadvantages regarding all these variables, except from unpaid overtime-work. Thus men tend, in general, to have more wage-promoting and time-consuming work characteristics compared with women.

**Table 1:** Descriptive statistics, mean/percentage SD in brackets

	Men	Women	Gender difference
Hourly wage (in SEK)	221.4 (132.9)	181.3 (77.4)	***
Prestige	45.5 (13.3)	44.9 (13.3)	*
Parent	69.4	78.9	***
Cohabiting/married	74.2	74.6	
Public sector	27.7	60.1	***
Years of education	13.2 (13.2)	13.5 (13.5)	*
Work exp.	21.6 (12.9)	20.7 (11.7)	*
% Female	30.8	65.8	***
Number of children in the household	0.9 (1.1)	1.0 (1.1)	
Child <12 years	33.0 (0.5)	34.0 (0.5)	
Housework (h)	8.3 (5.1)	13.5 (7.1)	***
*Time*-*consuming work*			
Working overtime	61.1	51.2	***
Unpaid overtime work	27.7	28.8	
Number of subordinates			
1–5	14.5	10.4	***
6–11	6.9	4.0	***
>11	10.1	5.7	***
Business travel (days)	12.2	3.5	***
*N*	2192	2082	

*** *p* = 0.001; ** *p* = 0.01; * *p* = 0.05

### The Gender Wage Gap by Family Status and Occupational Prestige

The first step is to analyze whether the gender wage gap varies with occupational prestige and family status i.e., between those employees who are (1) single, (2) part of a couple with children, and (3) part of a couple without children. It should be noted that because children are likely to have a long-term effect on wages—and particularly on women’s wages—“couples with children” also include couples with adult children. The analysis below is performed separately for the respective family categories and includes an interaction term for gender and occupational prestige. Consistent with previous research (Magnusson 2010), this interaction term is only statistically significant for couples with children (see Model 2 in Table 2). In other words, this result indicates that the gender wage gap among singles and cohabiting without children does not vary with the level of occupational prestige. Further, after taking occupational prestige into account we find no significant gender wage gap among singles. However, among cohabiting parents, the gender wage gap increases significantly with the occupational prestige level.^4^^,^ 5

**Table 2:** The gender wage gap by family status and occupational prestige

	Singles		Cohabiting, childless		Cohabiting, children	
	Model 1	Model 2	Model 1	Model 2	Model 1	Model 2
Women	−0.042	0.046	−0.102***	−0.153	−0.197***	0.165***
Prestige	0.011***	0.010***	0.010***	0.009***	0.012***	0.014***
Prestige × women		−0.001		0.002		−0.006***
Constant	4.649***	4.454	4.857***	4.753***	4.835***	4.432***
*R* ^*2*^	0.133	0.245	0.153	0.195	0.241	0.289
*N*	500	500	629	629	2551	2551

Standard errors are clustered at the individual level. Unstandardized coefficients from an OLS regression model

The models include controls for education, work experience, the proportion of women in the occupation, sector of employment

*** *p* = 0.001; ** *p* = 0.01; * *p* = 0.05

### Time-Consuming Job Characteristics and the Gender Wage Gap by Occupational Prestige

The second step in the analysis is to study the extent to which the above reported gender wage gap among couples with children is explained by gender differences with respect to access to the time-consuming and wage-promoting job characteristics discussed above. A previous analysis indicated that fathers have greater access than mothers to jobs with unpaid overtime, business travel and supervisory positions. The largest gender difference is related to business travel. Fathers’ also have more subordinates, and are to a greater extent expected to work overtime without extra pay more than mothers. Analysis is available upon request.

**Fig. 1:**
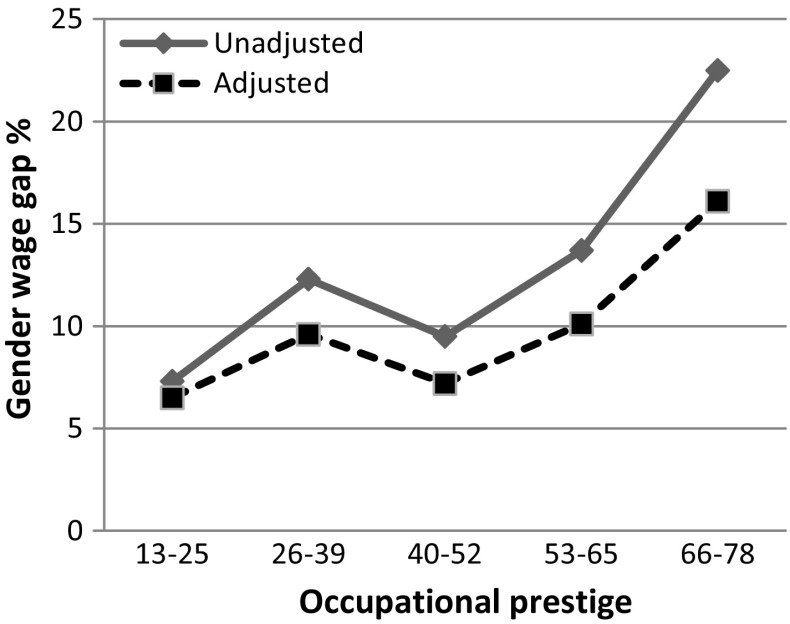
The association between the gender wage gap and time-consuming job characteristics. Only married/cohabiting parents included, n: 2551. *Note* The models include controls for education, work experience, the proportion of women in the occupation, sector of employment, housework, number of children, and young children in the household

The solid (unadjusted) line in Fig. 1 (based on estimates in Model 1 in Table 4 in “Appendix” calculated for a respondent with average values on all variables included in the analysis) represents the gender wage gap at different levels of occupational prestige, after controlling for level of education, work experience, housework, number of children in the household, young children, employment sector and the proportion of women in the occupation. Figure 1 shows that the gender wage gap in general increases with occupational prestige. It should be noted that the gender wage gap in occupations with the lowest prestige is not statistically significant. In high-prestige occupations (prestige level 66–78), the hourly wage for men is on average approximately 23 % higher than the hourly wage for women. After adding controls for access to employment featuring time-consuming job characteristics (dotted line in Fig. 1, based on estimates in Model 2, Table 4 in “Appendix”), the gender wage gap is significantly reduced, which is especially true in high-prestige occupations, in which the wage gap is reduced from 23 to 16 %. Thus, as expected, gender differences in access to these job characteristics are particularly important for explaining the gender wage gap in high-prestige occupations.

**Fig. 2:**
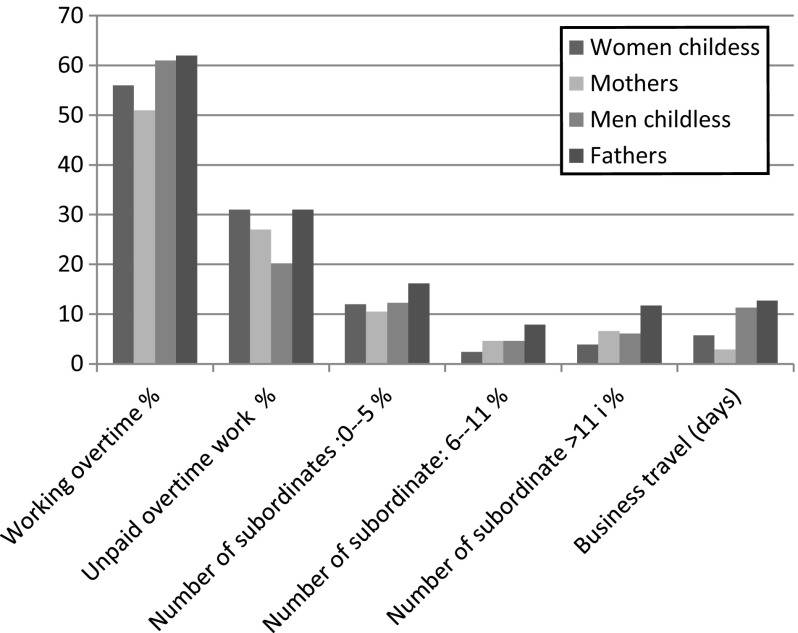
Distribution of working conditions by gender and family situation (among parents are only cohabiting parents included), n: 3680

The above analysis clearly indicates that gender differences in access to working conditions are difficult to reconcile with having the main responsibility for the family and are important for understanding the gender wage gap between mothers and fathers. Another fruitful question discussed above is whether mothers also have less access than childless women to these types of wage-promoting working conditions, in addition to asking whether fathers and childless men have equal access to these working conditions. To answer these questions, we perform separate analyses for men and women. Figure 2 below indicates relatively few statistically significant differences among women, i.e., between mothers and childless women, in access to these working conditions (for significance levels see Table 5 in “Appendix”). However, childless women tend to work more overtime and to have more business travel than mothers. Furthermore, it is somewhat more common for mothers to have a large number of subordinates (i.e., more than 6 subordinates).

However, Fig. 2 also shows that working conditions differ significantly between childless men and fathers. For instance, it is much more common for fathers than for childless men to have a job that requires high availability, i.e., a job that requires unpaid overtime. Fathers also have more subordinates than single men.

Taken together, the findings presented above indicate small differences between mothers and childless women regarding access to these working conditions. Instead, it seems as if fathers are particularly advantaged in terms of access to these types of wage-promoting working conditions. The third and final step in the analysis is therefore to study the extent to which fathers’ greater access to these working conditions accounts for the earlier reported fatherhood wage premium. However, first we investigate whether there is a wage gap between childless and cohabiting parents with separate analysis for men respectively women, and whether this potential wage difference varies with occupational prestige.

Table 3, model 1 indicates that being a parent is positively related to wage for men. For women, these relationships are reversed, and there is a negative association between being a parent and wage. Among both men and women, as measured within gender, we find a clear wage premium for being married or cohabiting. In model 2, we add an interaction term between being a parent and occupational prestige to investigate the extent to which the wage premium of being a father varies with the level of occupational prestige. The positive and significant estimate of the interaction term among men indicates that the fatherhood wage premium increases with occupational prestige. Thus, the results show a difference between fathers and non-fathers in wage return per increment in occupational prestige, where non-fathers get a lower increase in wages than do men with children. We find no corresponding significant association among women. However, an analysis not presented indicates that the negative association between being a parent and wage is only significant among women in rather low prestigious occupations, i.e., occupational prestige below 45 (results available upon request).

**Table 3:** Within-gender association between parenthood and wage

	Women		Men	
	Model 1	Model 2	Model 1	Model 2
Parent	−0.080***	−0.031	0.072**	−0.108
Prestige	0.010***	0.010***	0.012***	0.009***
Parent × prestige		-0.001		0.004**
Married/cohabiting	0.041**	0.041**	0.069***	0.074***
Constant	4.672***	4.637***	4.340***	4.459***
*R* ^*2*^	0.234	0.235	0.259	0.263
*N*	2082	2082	2192	2192

Separate models for men and women. Standard error clustered at the individual level. Unstandardized coefficients from an OLS regression model

The models include controls for education, work experience, age, the proportion of women in the occupation, sector, young children in the household and housework

*** *p* < 0.001; ** *p* < 0.01; * *p* < 0.05

**Fig. 3:**
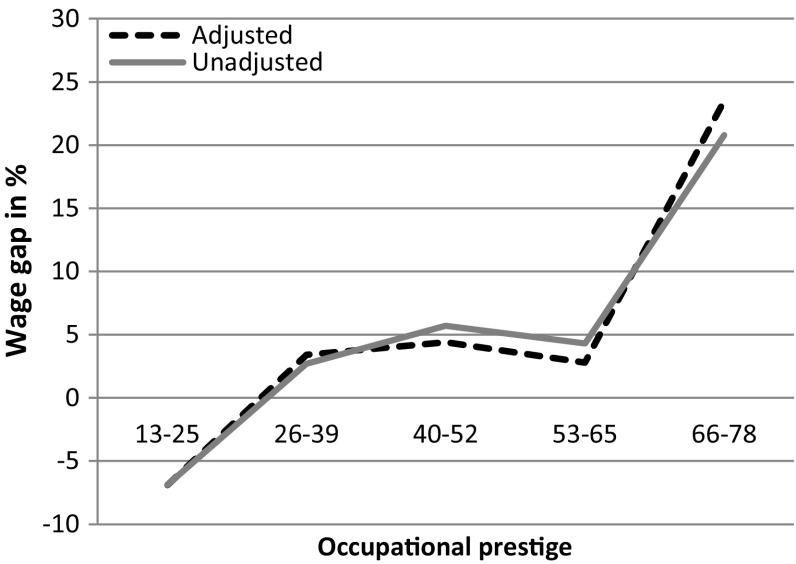
The association between wage and parenthood. Men only (n: 2192). Standard errors are clustered at the individual level. *Note* The models include controls for education, work experience, age, the proportion of women in the occupation and sector

In Fig. 3 (based on estimates in Table 6 in “Appendix”), we analyze the wage gap between fathers and single childless men at different levels of occupational prestige. The solid line (unadjusted) shows that the wage gap between fathers and childless men in high-prestige occupations is quite substantial. However, the wage premium for parenthood is only statistically significant in high-prestige occupations (66–78 prestige). When controls for time-consuming working conditions are introduced, the dotted line (adjusted), the positive coefficient for being a parent increases in high-prestige occupations. Thus, the fatherhood wage premium is not explained by differences in time-consuming working conditions. Instead, fathers are even more advantaged in terms of wages when accounting for their access to these time-consuming working conditions.

## Discussion

One purpose of this paper was to study the role of family status and time-consuming working conditions to better understand the gender wage gap at different levels of occupational prestige. Several conclusions can be drawn from our findings. First, separate analyses of (1) single persons, (2) couples with children and (3) couples without children indicate that the gender wage gap is smallest among single women and men, and largest among those cohabiting with children. However, it is notable that the average wage is highest among couples with children. Second, the analyses clearly indicate that the gender wage gap increases with the level of occupational prestige, which is consistent with previous research (cf. Magnusson 2010). Perhaps of greater interest is that this association is significant only among cohabiting men and women with children. Thus, we find no evidence that the gender wage gap varies by occupational prestige among single persons and childless cohabiting individuals. Further, given the same occupational prestige, we find no significant gender wage gap among singles. The analysis presented herein also shows that gender differences in access to jobs with time-consuming characteristics reduce the gender wage gap significantly and particularly so in high-prestige occupations. Thus, the gender wage gap between mothers and fathers is partially explained by the fact that mothers are less likely to have jobs that contain wage-promoting and time-consuming working conditions.

A second purpose here was to test whether there is a within-gender association between parenthood, job characteristics and wage and, if so, to determine the extent to which this parent premium or penalty varies with occupational prestige. Our findings indicate that there is a marriage premium for both men and women, although parenthood only seems to be positively related to wages for men. However, this fatherhood premium is only statistically significant in high-prestige occupations. The analysis indicates that fathers are more advantaged than non-fathers both with regard to wage and access to jobs characterized by time-consuming conditions. However, in contrast to the analysis of the wage gap between mothers and fathers, the wage gap between fathers and childless men increases after controlling for these job characteristics. Thus, adjusting for fathers’ greater access to jobs characterized by these working conditions further increases the wage gap among men in high-skilled occupations.

Worth to remembering is that the lower amount of time-consuming work does not only apply to mothers but also to non-mothers. In general the findings indicate rather small differences between mothers and non-mothers, both in terms of wages and in terms of time-consuming work. This is a divergence from prior studies regarding motherhood and wages in America which report a negative relationship between motherhood and wages (e.g., Budig and Hodges 2010; Gough and Noonan 2013). However, other prior studies from the Nordic countries point in similar direction and find weak or none relationship between motherhood and wages (Gash 2009; Petersen et al. 2014). Some prior studies even show a positive association between motherhood and wages in Sweden (Bihagen et al. 2014b; Boschini et al. 2011). This may indicate that the motherhood wage penalty is smaller or even absent in countries that support maternal employment (Gash 2009) but the wage gap between men and women is at the same time present and large.

There are no self-evident explanations for why women and childless men have lower access than fathers to jobs with these types of wage-promoting job characteristics. Previous research suggests that these job characteristics are more common in male- than in female-dominated occupations (see e.g., Presser and Hermsen 1996). However, controlling for the proportion of women in the occupation and for the sector of employment does not change our findings. Furthermore, results from separate analyses for private and public sector point in similar directions, thus the gender wage gap in both private and public sector increases significantly with the occupational prestige level (analyses available upon request). Consequently, we find no empirical evidence to explain gender differences in working conditions by the fact that men and women work in different occupations and sectors. This finding is further supported by a Swedish study reporting that time-consuming work characteristics are actually most common in gender-integrated occupations and that overtime work is more common in female- than in male-dominated occupations (Kjellsson et al. 2014).

A drawback with the present study is that we cannot tell whether the gender differences in time-consuming characteristics are due to gender differences in preferences, productivity or due to different treatment of mothers and fathers by the employers (or have other explanations). A possible explanation could be that mothers choose to abstain from such working conditions to enable the combination of work and family. However, the reported rather small difference between mothers and non-mothers regarding these time-consuming working conditions speaks against this explanation. Nevertheless, more research is needed to determine whether the fatherhood premium in high-prestige occupations, in particular, is due to self-selection, preferences or the fact that employers treat men and women differently. It is possible that fathers are offered positions characterized by this type of wage-promoting and time-consuming working conditions more often than mothers. Because women continue to have, or are deemed to have, the main responsibility for the family—not least because of the uneven distribution of parental leave days—employers may perceive them as having more difficulties than men with handling time-consuming work. If so, employers may be inclined to choose a man before a woman to undertake certain tasks and positions, which in turn leads to fewer career opportunities for women.

Fathers are, however, not only more advantaged than women in general but also more advantaged than single childless men—particularly in high-prestige occupations. This finding is consistent with previous research showing that the fatherhood premium increases along the earnings distribution (e.g., Prince Cooke 2014) and with Hodges and Budig’s (2010) result of a larger fatherhood bonus among highly educated and privileged men. In this context, cultural perceptions of what constitutes a promising and competent employee may also be a possible explanation. Compared with a single man, a married father may be considered more stable and reliable by employers (cf. Hodges and Budig 2010). The positive association between parenthood and labor market outcomes might of course also be due to selection. Thus, more “successful” men are relatively more likely than other men both to marry and have children (see e.g., Ludwig and Brüderl 2011). However, it should be noted that the fatherhood wage premium is highest in high-prestige occupations in which both the fathers and the childless men are highly skilled and highly paid men.

Overall, the presented findings are in line with previous research indicating a bonus for parenthood and marriage among men (e.g., Killewald 2013; Petersen et al. 2014; Prince Cooke 2014), and that this bonus is important for understanding the overall gender wage gap (Petersen et al. 2014). The findings also clearly show that the association varies considerably by occupational prestige which point out the importance of taking level of occupational prestige into account when analyzing the relationship between family status and wages.

## Appendix

See Tables 4, 5 and 6.

**Table 4:** The association between the gender wage gap and time-consuming job characteristics

Prestige level	13–25	26–39	40–52	53–65	66–78
*Model 1*					
Women	−0.072	−0.123**	−0.095**	−0.137***	0.225***
Work exp.	0.024**	0.010*	0.026***	0.033***	0.027
Work exp.^2^	−0.001***	−0.000*	−0.001***	−0.001***	−0.001
Education	−0.010	−0.007	0.018***	0.021***	0.034**
Sector (public)	0.048	0.026	−0.057*	−0.173***	−0.074
% Female in occ.	−0.002	−0.001*	−0.002***	−0.004***	−0.004
Housework	−0.002	0.004	−0.002	−0.004	0.009
Number. children	−0.015	−0.038**	−0.010	−0.030	−0.091**
Young children	0.081	0.011	0.048	0.029	−0.157
Constant	5.001***	5.272***	4.920***	5.178***	5.308***
R^2^	0.113	0.086	0.154	0.226	0.187
*Model 2*					
Women	−0.065	−0.096*	−0.072*	−0.101**	−0.161**
Work exp.	0.023***	0.007	0.024***	0.026***	0.028*
Work exp.^2^	−0.001***	−0.000	−0.001***	−0.001***	−0.001*
Education	−0.010	−0.009	0.011**	0.013**	0.016
Sector (public)	0.054	0.018	−0.013	−0.145***	−0.072
% Female in occ.	−0.001	−0.001*	−0.002***	−0.002**	−0.138
Housework	−0.003	0.004*	−0.002	0.000	0.000
Number. children	−0.014	−0.034**	−0.017	−0.043*	−0.138
Young children	0.084	0.016	0.045	0.044	−0.032
Working overtime	0.018	0.068**	0.107**	0.089***	0.170**
Unpaid overtime work	0.028	−0.015	0.057***	0.026	0.101
Number of subordinates					
1–5	0.093	0.038	0.078*	−0.098**	0.080
6–11	0.161	0.072	0.105*	0.147**	−0.075
>11	0.331***	0.012	0.159***	0.305***	−0.330***
Business travel (days)	0.001	0.002	0.002***	0.003*	0.006*
Constant	4.969***	5.240***	4.923***	5.076***	5.119***
*R* ^*2*^	0.145	0.145	0.228	0.337	0.429
*N*	216	515	959	662	198

Only for married/cohabiting parents (n: 2551) Standard errors are clustered at the individual level. Unstandardized coefficients from an OLS regression model

*** *p* < 0.001; ** *p* < 0.01; * *p* < 0.05

**Table 5:** Distribution of time-consuming work variables by gender and family situation

	Women			Men		
	Parents	Singles	Diff.	Parents	Singles	Diff.
Working overtime	27	36	*	38	36	
Unpaid overtime work	27	31		31	20	***
Number of subordinates						
1-5	10.5	12		16.2	12.3	*
6-11	4.6	2.4	*	7.9	4.6	*
>11	6.6	3.9	*	11.7	6.1	***
Business travel (days)	2.9 (10.1)	5.7 (22.8)	***	12.7 (33.2)	11.3 (31.3)	
*N*	1281	460		1270	669	

Among parents are only cohabitation parents included. Mean/percentage SD in brackets

*** *p* = 0.001; ** *p* = 0.01; * *p* = 0.05

**Table 6:** The association between wage and parenthood

Prestige level	13–25	26–39	40–52	53–65	66–78
*Model 1*					
Parent	−0.069	0.027	0.057	0.043	0.208***
Married	0.129*	0.081**	0.022	0.117*	0.010
Work exp.	0.030**	0.016**	0.020**	0.029**	0.024
Work exp.^2^	−0.001*	−0.000**	−0.000	−0.001***	−0.001
Education	0.002	−0.013*	0.030***	0.028**	0.026
Sector (public)	0.058	0.024	−0.147***	−0.191***	−0.013
% female in occ.	−0.000	−0.002***	−0.000	−0.005***	−0.005
Age	−0.010	−0.001	0.001	0.003	0.015
Constant	4.930	5.192	4.727	4.857	4.427
R^2^	0.149	0.108	0.098	0.188	0.194
*Model 2*					
Parent	−0.069	0.034	0.044	0.028	0.235***
Married	0.126	0.074***	0.009	0.139**	0.022
Work exp.	0.030*	0.009	0.015*	0.019*	0.009
Work exp.^2^	−0.001	−0.000	−0.000	−0.001**	0.000
Education	0.003	−0.017**	0.019**	0.017*	0.013
Sector (public)	0.063	−0.005	−0.089**	−0.129**	−0.047
% female in occ.	−0.000	−0.002**	−0.001*	−0.003**	0.004
Age	−0.010	0.003	0.002	0.004	0.017
Working overtime	0.012	0.053*	0.093***	0.152***	0.261***
Unpaid overtime work	0.040	−0.054	0.170***	0.088*	0.070
Number of subordinates					
1–5	0.080	0.019	0.071	0.080	0.045
6–11	0.158	0.121*	0.120**	0.122	0.010
>11	0.522	0.266***	0.177***	0.208***	0.388
Business travel (days)	0.001	0.001**	0.002*	0.002	0.003
Constant	4.891***	4.891***	4.765***	4.733***	4.142***
*R* ^2^	0.206	0.160	0.211	0.290	0.406
*N*	124	642	807	414	204

Men only (n: 2192). Standard errors are clustered at the individual level. Unstandardized coefficients from an OLS regression model

*** *p* < 0.001; ** *p* < 0.01; * *p* < 0.05

## Footnotes

1 Killewald (2013) only find a fatherhood wage premium among married residential biological fathers.

2 However, one problem regarding marriage and fatherhood wage premium is that it is often difficult to determine cause and effect. From a selection perspective, the male wage premium for marriage and parenthood is due to differences in productivity and personnel characteristics between men who get married and/or are parents and those who remain unmarried without children (e.g., Ludwig and Brüderl 2011. Thus, characteristics rewarded on the marriage market are also rewarded in the labor market. Conversely, the treatment hypothesis posits that men and women change their behavior when they marry, and men, in particular, tend to increase their employment effort and productivity (e.g., Petersen et al. 2014).

3 Separate analyses for year 2000 and 2010 have also been done as a sensitivity test, when possible. Overall, the findings are in line with the findings presented here. However, the separate analyses indicate to some extent that time-consuming work explain a larger part of the gender wage gap year 2000 than 2010 (results available upon request).

4 All models have also been carried out with a control for year 2000 and with a control for part-time work. The results are not substantially different to those reported here.

5 Estimations with separate models for the private and public sector have also been made with similar results. Thus, for both private and public sector the gender wage gap increases significantly with the occupational prestige level.
